# On Local Anæsthetic—Cocaine

**Published:** 1885-11

**Authors:** C. C. Burns

**Affiliations:** Greensburg, Indiana


### CORRESPONDENCE.

### On Local Anæsthetic—Cocaine.

EDITOR DENTAL REGISTER—

*Dear Sir:*—It is not my intention to criticise nor cast reflection on the judgment of those of our profession who have so willingly and with much pains and trouble endeavored to enlighten us on this subject. The modus operandi and the soothing effects which *cocaine* seems to possess in the hands of some, seem so marvelous to me, that I sometimes wonder if it can be *possible* that I am not competent to appreciate the wonderful effects it does produce on the human economy. It may be that my imagination is not developed sufficiently to realize the soothing power contained in this drug, *for dental purposes.* I have been a dental practitioner for twenty-nine years, and I believe in progression, at the same time I feel that I am left behind, when I see the advancement made by younger men who have not had one-third of that experience. I am satisfied that cocaine may be used advantageously in some operations on the human economy, but not to advantage on teeth and the mucous membrane of the mouth. I am sorry to say that as far as my experience goes in the use of cocaine, the results are far from being satisfactory; I am aware that imagination goes a great way in the extracting of teeth, localities may have something to do with the soothing effects this cocaine produces on the imagination of the patient, and more especially the operator who cries eureka.

I am in for painless operations, and so are the people, who sooner or later are compelled to submit to the dental surgeon, but we want facts, not fancies. When I say facts I don't mean to cast any reflection on those who have started out to do so, but have failed in their undertaking. Had they given us the number of their failures their little success would be left in the shade. As far as my experience goes in the use of cocaine for dental purposes, it is a total failure. I have tried it on a great number of persons for extracting, and also for devitalizing sensitive dentine, and but very little benefit did I derive from its use, but I was determined to test it thoroughly for my own satisfaction, and as I had a molar tooth that had given me considerable trouble, I came to the conclusion that I was a fit subject to judge of its merits and its demerits. I therefore had my gums treated hypordermically, and also bathed in the solution of cocaine for twenty-five minutes. The tooth was finally extracted. What was the result? I was like the little boy. Just before I died the tooth came out. Now, after twenty-nine years of successful practice in dental surgery, can it be possible that I know so little. Are my perceptive faculties so deficient that I am unable to perceive the *eureka* that is contained in cocaine? May it not be possible, however, that there are other old practitioners that we may hear from, who will give a true statement as I have already done, in regard to their success in the use of this drug as an anæsthetic. It is not my desire, nor aim, to place myself before the Dental Profession as an expert. But should I be called upon to fill the chair, I will do so with pleasure, and show to those who are seeking after facts, that I have been unable to find the "mare's nest," yet such a nest surely exists.

Respectfully,

C. C. Burns.

Greensburg, Indiana.

